# Interaction between intestinal flora and gastric cancer in tumor microenvironment

**DOI:** 10.3389/fonc.2024.1402483

**Published:** 2024-05-21

**Authors:** Mingjin Yang

**Affiliations:** Department of Gastrointestinal Surgery, The Affiliated People’s Hospital of Ningbo University, Ningbo, China

**Keywords:** gastric cancer, tumor microenvironment, bacterial extracellular vesicle, antitumor drugs, cancer

## Abstract

Gastric Cancer (GC) is a prevalent malignancy globally and is the third leading cause of cancer-related deaths. Recent researches focused on the correlation between intestinal flora and GC. Studies indicate that bacteria can influence the development of gastrointestinal tumors by releasing bacterial extracellular vesicles (BEVs). The Tumor microenvironment (TME) plays an important role in tumor survival, with the interaction between intestinal flora, BEVs, and TME directly impacting tumor progression. Moreover, recent studies have demonstrated that intestinal microflora and BEVs can modify TME to enhance the effectiveness of antitumor drugs. This review article provides an overview and comparison of the biological targets through which the intestinal microbiome regulates TME, laying the groundwork for potential applications in tumor diagnosis, treatment, and prognosis.

## 1 Introduction

GC is the fifth most commonly diagnosed cancer worldwide and the third leading cause of cancer death globally (1). According to the latest estimates from GLOBOCAN, there are nearly 1.1 million new cases of GC each year, with approximately 800,000 deaths attributed to the disease annually, representing about 7.7% of all cancer-related deaths (2). Risk factors for GC include age, social status, lifestyle, heredity, surgical history, and diet. Interestingly, the incidence of GC is rising among individuals under 50 in both low and high risk regions, possibly due to the increasing prevalence of obesity and disruptions in the gastric microbiome associated with modern lifestyles (3). Surgical resection is the recommended treatment for early GC, while chemotherapy is the primary option for patients who are not eligible for surgery or have advanced metastatic disease (4). However, drug resistance, whether inherent or acquired, often leads to poor or no response to chemotherapy in GC patients, posing a significant challenge in treatment outcomes (5). Despite advancements in targeting known pathways in GC treatment, obstacles remain, such as limited drugs targeting specific pathways and restricted applicability due to the high heterogeneity of the disease. Therefore, there is an urgent need to investigate new mechanisms and potential therapeutic targets for GC.

In recent years, there has been a growing focus on the relationship between the gut microbiome and GC (6). The human gut microbiome is comprised of trillions of microorganisms, primarily bacteria, fungi, protozoa, archaea, and viruses (7). BEVs produced by intestinal symbiotic bacteria, probiotics, and pathogenic bacteria have been discovered to influence the intestinal microenvironment and overall host health. Changes in the composition of intestinal microorganisms and their secreted BEVs can significantly impact the development and progression of GC (8). TEM consists of immune cells and stromal cells with both immunosuppressive and immunogenic cytokines. The immune cells include T cells, B cells, NK cells, macrophages, neutrophils, dendritic cells. The stromal cells include endothelial cells, cancer associated fibroblasts, adipocytes, stellate cells. The interaction between intestinal microbiota and their secreted BEVs with TME can directly lead to TME reprogramming and have profound effects on tumor immunity (9). While intestinal bacteria are vital for regulating the intestinal microenvironment and host health, the specific mechanisms underlying this regulation remain largely unexplored. This article delves into the impact of intestinal microbiota and BEVs on TME, as well as the potential mechanisms influencing tumor initiation and progression. Furthermore, the therapeutic potential of BEVs in the context of tumors is also discussed.

## 2 Intestinal flora and stomach cancer

### 2.1 Intestinal flora

Intestinal flora, a complex community of a variety of microorganisms including bacteria, fungi, protozoa, archaea, and viruses, plays an important role in maintaining homeostasis (7). The acquisition of gut microbiota begins at birth and stabilizes around 3 years of age in humans and 8 weeks in mice (10). Approximately 30–40 bacterial species dominate the adult flora, with *Bacteroides, Bifidobacterium, Eubacterium, Clostridium, and Lactobacillus* being prevalent (11). Intestinal flora contributes to host energy metabolism, nutritional balance, and immune regulation. It helps in resisting pathogen invasion by secreting antibacterial peptides, stimulating immune cells, producing antibodies, and promoting T-cell differentiation (12). Moreover, it aids in preventing colonization of pathogenic bacteria by inducing IgA secretion, producing antibacterial substances, and regulating tight junction integrity (13). Additionally, intestinal flora can facilitate epithelial healing, as evidenced by reduced intestinal mucosa damage from cisplatin treatment through fecal gavage (14).

*Helicobacter pylori (H. pylori)* is a microaerobic, helical, flagellated Gram-negative bacterium that colonizes the human gastric mucosa (15). Urease and adhesin produced by *H. pylori* assist the bacteria in colonizing and surviving in the harsh stomach environment (15). The majority of GC cases caused by *H. pylori* are of the intestinal type non-cardiac GC variety, following a predictable progression from atrophic gastritis to intestinal metaplasia, dysplasia, and ultimately GC (16). The distribution of the gastric microbiome in the development of GC is still largely unclear. Therefore, it is crucial to investigate the components of the gastric microbiome and the specific bacteria involved in the pathogenesis of GC to develop potential prevention and treatment strategies.

### 2.2 Development of GC and intestinal flora

#### 2.2.1 Intestinal flora and precancerous lesions

Recent studies have focused on investigating changes in gastric microbiota in patients with chronic gastritis or precancerous lesions such as atrophy and intestinal metaplasia. These studies aim to determine if alterations in gastric microbiota are associated with the development of GC. Atrophic gastritis is primarily caused by the prolonged presence of *H. pylori* infection, leading to the destruction of acid-secreting cells and an increase in stomach pH. Consequently, this environment allows bacteria from the oral cavity and duodenum to survive in the stomach.

In a 2009 study (17), researchers investigated the DNA-based bacterial community composition in the stomachs of GC patients using 16S rRNA gene cloning and sequencing. The gastric microbiota of GC patients and dyspeptic controls were found to be dominated by *Firmicutes* and oral taxa such as *Streptococcus, Lactobacillus, and various Clostridium genera*. However, due to a limited number of subjects, no significant differences in microbial composition were observed between cancer patients and controls. Subsequently, a 2016 large-scale study focused on 212 patients with chronic gastritis and 103 patients with GC to characterize the gastric microbiota. The authors used quantitative PCR to determine that the bacterial load of gastric mucosa in GC patients was significantly higher than in chronic gastritis patients (18). Furthermore, the biodiversity and composition of the gastric microbiota in a subset of patients were analyzed by pyrosequencing, revealing no significant difference in diversity index between GC patients and chronic gastritis patients. Nevertheless, enrichment of five bacterial genera (*Lactobacillus, Shigella, Nitrospirochete, Burkholderia, and Spirulina lagardi*) was observed in GC.

In a study conducted in Portugal (19), 54 GC patients and 81 chronic gastritis patients underwent examination through 16S rRNA sequencing. The findings revealed that the GC microbiota exhibited reduced microbial diversity, lower levels of *H.pylori*, and higher proportions of *achromobacter, citrobacter, phyllobacter, clostridium, rhodococcus, and lactobacillus*. Furthermore, the authors utilized bioinformatics software PICRUSt to predict an increase in nitrosation-related bacterial abundance in GC patients.

In a study conducted by Chinese researchers (20), the gastric microbiome of 81 patients with gastritis and GC was analyzed using 16S rRNA sequencing. The results revealed significant dysregulation in the microbiome of GC patients, characterized by an enrichment of *Parvimonas micra, Dialister pneumosintes, Slackia exigua, Peptostreptococcus stomatis, Prevotella intermedia, Fusobacterium nucleatum, Prevotella oris* and *Catonella morbi* compared to patients in the precancerous stage. Furthermore, the GC microbiome showed a significant increase in operational taxonomic units (OTUs) corresponding to *Prevotella intermedia, Fusobacterium nucleatum, Prevotella oris*, and *Catonella morbi*. A separate study in Singapore and Malaysia supported these findings, suggesting that microbial factors beyond *H. pylori* may contribute to the development of GC. Specifically, *Lactococcus, Velociella*, and *Clostridiaceae bacteria* were found to be dominant in GC patients and those with dyspepsia (21).

In comparison to previous research, it has been discovered that GC is linked to an increased diversity and richness of the microbiota. Chang et al. conducted a study comparing the gastric microbiota of 31 Korean patients, which included 11 patients with non-cardiac GC, 10 patients with intestinal metaplasia, and 10 patients with chronic gastritis. The study revealed significant variations in the gastric microbiota of patients dominated by *H.pylori*. Among the bacterial groups that were more abundant in GC patients were *Streptococcus, Lactobacillus, Veillonella, and Prevotella (* 22).

According to the Correa cascade reaction, GC has evolved through multiple stages influenced by environmental and genetic factors, including superficial gastritis (SG), chronic atrophic gastritis (CAG), intestinal metaplasia (IM), dysplasia (DYS), and GC (22, 23). A study in Colombia found that *Leptotrichia wadei, Veillonella* sp., and *Streptococcus* spp. were more abundant in CAG subjects with IM (24). In high-risk areas of GC in China, there was a significant increase in the incidence of *H.pylori, Clostridium, Neisseria, Porphyromonas, Prevotella, Rossiella*, and *Velociella* in the advanced gastric disease group (CAG/IM/DYS) compared with the normal/superficial gastritis (SG) group (25). Conversely, another study revealed that *Bifidobacterium and Klebsiella* were enriched in SG compared to subjects with more severe lesions (26). When IM or dysplasia (DYS) were considered severe precancerous lesions, *Pseudomonas and Dyella* were significantly overrepresented in IM compared with SG subjects (20). Metagenome sequencing analysis showed that commonly thought oral pathogens *Johnsonella ignava* and *Filifactor alocis* were positively correlated with IM, while *Streptococcus mutans, Streptococcus parasumoides*, and *Streptococcus sanguis* were negatively correlated with IM (27). Prospective observations also indicated enrichment of *phyllobacteriaceae, Enhydrobacte*r, and *Moryella genera*, and depletion of *Bifidobacterium* and *Lactobacillus genera* in early GC. *Actinobacteria* were increased in IM patients at high risk for gastric disease progression. The concentrations of *Sphingomonas* and *Acinetobacter* in GIN patients were higher than those in mild or moderate gastric lesions (26, 28).

In addition to precancerous lesions and cancer, gastric microflora may also be associated with non-ulcerative dyspepsia and gastric ulcers. For instance, *H.pylori, Prevotella, Neisseria*, and *Streptococcus* are more common in patients with gastric ulcers, whereas patients with dyspepsia often exhibit gastric microflora characterized by *Propionibacterium, Lactobacillus, Streptococcus*, and *Staphylococcus (* 21, 29). Moreover, several studies have noted variations in gastric microbiota composition between GC patients and those with gastritis. Although these studies have not reached unanimous conclusions, they have established differences in gastric microbiota between GC patients and those without GC. Further research is necessary to fully comprehend these findings.

### 2.3 Intestinal flora and GC

#### 2.3.1 *H. pylori* and GC

**Figure 1:**
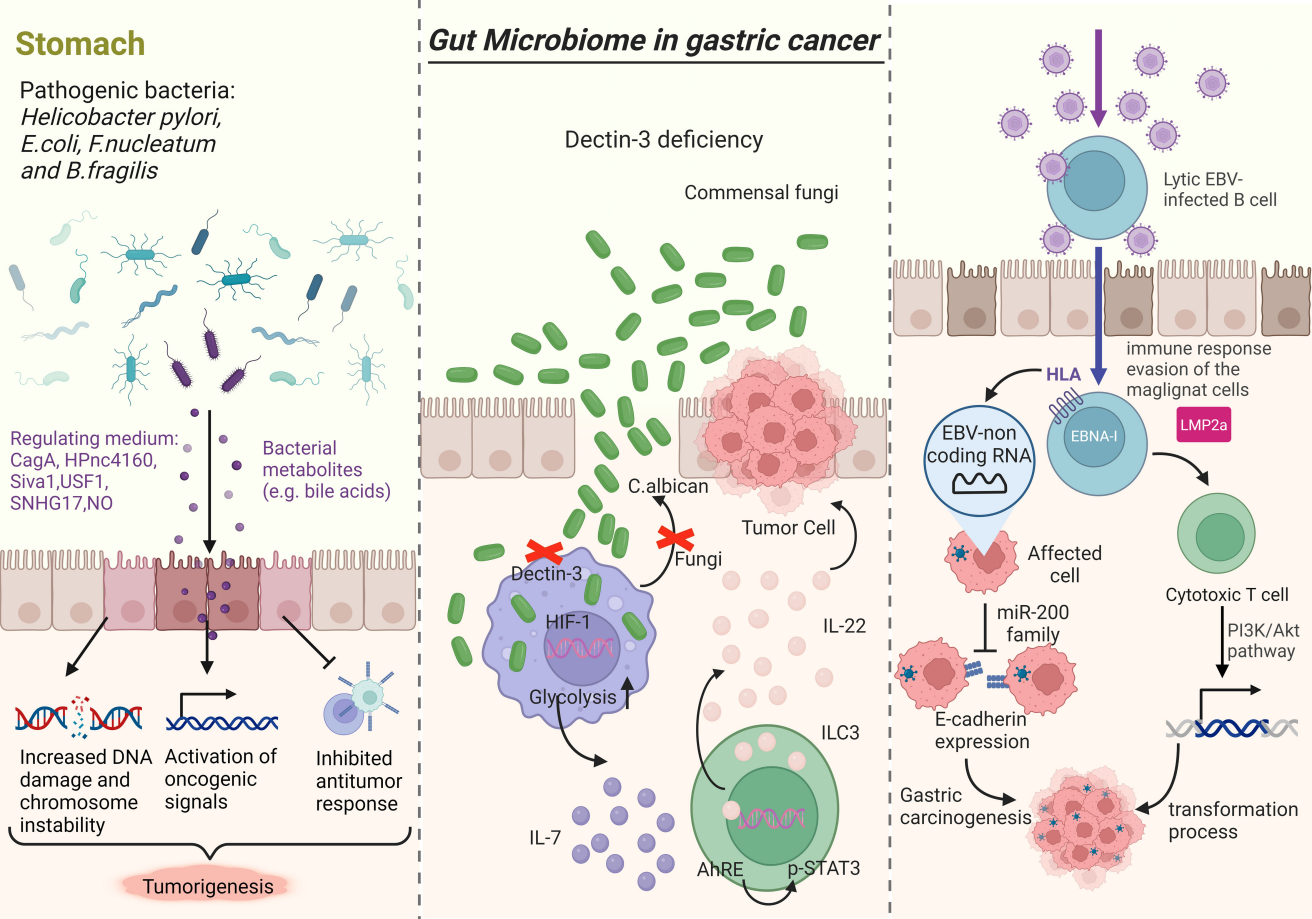
Research indicates that gut microbiota disorders play a role in the progression of GC. The left picture illustrates how bacterial pathogens like *H. pylori, E. coli, F. nucleatum*, and *B. fragilis* can promote tumorigenesis by releasing virulence factors and toxins that increase DNA damage, chromosomal instability, activate oncogenic signals, and suppress immune responses. *Fungi*, particularly *C*. *albicans*, can impact carcinogenesis by affecting macrophage function and glycolysis levels, leading to increased IL-7 secretion and subsequent effects on transcription factors and cytokine production (middle picture). EBV-non coding RNA (EBERS) is linked to the downregulation of the miR-200 family, resulting in decreased E-cadherin expression (right picture).

The relationship between intestinal microbiome dysregulation and GC has garnered increasing attention from researchers (**Figure 1**). It is well established that *H. pylori* infection is a major risk factor for intestinal GC, with the International Agency for Research on Cancer classifying it as a class I carcinogen as early as 1994 (30). Animal infection models have shown that *H. pylori* can induce GC in Mongolian gerbils, closely resembling the Correa model of intestinal GC (31, 32). Infection with *H. pylori* leads to chronic inflammation of the gastric mucosa, destruction of oxytocin cells, an increase in stomach pH, and an imbalance in the gastric microbiota, resulting in decreased *H. pylori* levels and colonization by non-*H. pylori* bacteria. This stage is characterized by the simultaneous promotion of reactive nitrogen oxide production by *H. pylori* and chronic inflammation, leading to DNA damage, apoptosis, and autophagy of gastric epithelial cells, ultimately resulting in gastric mucosal damage and the development of *H. pylori*-related gastropathy and GC (33). Mechanisms linking *H. pylori* infection to gastric carcinogenesis include colonization of *H. pylori* in the gastric mucosa, toxin-induced damage to the gastric mucosa, biological immune responses, and chronic inflammation (29).

The response to *H. pylori* infection is primarily driven by a variety of virulence factors such as urease, vacuolating cytotoxin A (VacA), cag pathogenicity island, cytotoxin-associated gene A (CagA), peptidoglycan outer membrane proteins (e.g. BabA, SabA, OipA), and gamma-glutamyl transferase (GGT). CagA, in particular, plays an important role in the inflammatory response and carcinogenesis triggered by *H. pylori*. Upon translocation into gastric epithelial cells, CagA activates extracellular signal-regulated kinases (ERK), a known oncoprotein that disrupts various signaling pathways and promotes malignant transformation of host cells (34, 35).

In April 2021, a Japanese research team published their findings on HPnc 4160, a small RNA molecule in *H. pylori* that can inhibit the expression of CagA and outer membrane protein (OMP). This inhibition helps protect *H. pylori* and allows it to better adapt to extreme environments (36). Autophagy is a cellular mechanism that eukaryotic cells use to maintain homeostasis. Studies suggest that the Hp virulence factor CagA may trigger the degradation of the pro-apoptotic gene Siva1 through the regulation of the PI 3K/Akt signaling pathway, leading to the inhibition of autophagy. This inhibition allows cells with DNA damage to persist, ultimately leading to malignant cell transformation (37). T cell responses play an important role in the development of inflammation after *H. pylori* (Hp) infection. Various virulence factors have been identified to regulate the intensity of the inflammatory response, which can hinder T cell activation, decrease phagocytosis, or aid in evading toll-like receptor (TLR) recognition. One such factor is the secreted vacuolar toxin VacA (38).

In September 2020, a study published in Gut magazine validated the significant protective role of the USF1 gene in Hp carcinogenesis across human, animal, and cellular levels. The study revealed that Hp infection leads to the degradation of p53 through the down-regulation of USF1 expression, ultimately causing DNA damage and GC. The findings suggest that USF1 could potentially serve as a marker for susceptibility to GC (39). Chinese scholars have investigated the role of long-chain non-coding RNAs (lncRNAs) in *H. pylori* (Hp) carcinogenesis. They have discovered that Hp infection can increase the expression of lncRNA SNHG17, leading to the disruption of the DNA damage repair system via the SNHG17/NONO and SNHG17/miR-3909/RING1/Rad51 signaling pathways. This ultimately results in genome instability and facilitates the transformation of inflammation into cancer (40). Another study from Japan discovered that chronic inflammation of the gastric mucosa leads to the production of high levels of nitric oxide (NO), resulting in alterations to transcriptional regulation in gastric cells through the upregulation of DNA methyltransferase activity. Inflammation caused by Hp infection generates reactive oxygen species (ROS), causing tissue damage and elevating oxidative stress in the stomach. This process can lead to DNA mutations in gastric cells, ultimately contributing to the formation of tumors (41). It is recognized that not all individuals infected with *H. pylori* will develop GC (42). Research indicates that genetic variations in inflammatory factors and other environmental factors, particularly dietary factors like high salt intake, iron deficiency, and nitroso compounds, play a role in increasing the risk of GC post-*H. pylori* infection (43, 44). Various studies have highlighted the role of nitrate-reducing bacterial species, including *H. pylori, Clostridium, Veillonella, Haemophilus, Staphylococcus, Neisseria, Lactobacillus, Escherichia coli*, and *nitrospirochete*, in promoting the malignant transformation of GC (29). Prospective cohort studies have demonstrated that the eradication of *H. pylori* infection significantly lowers the risk of GC, especially when done before the occurrence of intestinal metaplasia in the gastric mucosa (45–47). Recent research has also shown that *H. pylori* eradication not only reduces the risk of GC in asymptomatic patients but also decreases the risk of metachronous cancer after endoscopic resection in early GC patients (34, 35). Eradication of *H. pylori* can lead to changes in the composition of intestinal flora, and the use of probiotics can help restore the balance of intestinal flora disrupted by antibiotics in *H. pylori* eradication protocols (48, 49).

#### 2.3.2 Other microorganisms in stomach and GC

In addition to *H. pylori*, other microorganisms in the stomach have been linked to GC (50). Research has shown that there is an increase in the abundance of bacteria in the stomach and a decrease in diversity as the pathological process progresses from chronic non-atrophic gastritis to GC (19–22, 51, 52). Furthermore, there is a notable imbalance of bacteria in the stomach of GC patients. Specific bacteria such as *Peptostreptococcus stomatis, Streptococcus anginosus, Parvimonas mira, Slackia exigua*, and *Dialist pneumosintes* have been suggested to be associated with GC, but further experiments are required to confirm this (20). Another study discovered that *Acrobacter, Citrobacter, Phyllobacterium, Clostridium, Rhodococcus*, and *Lactobacillus* were more abundant in stomach cancer patients compared to those with chronic gastritis (19). Metagenomic function studies of GC-related flora revealed a significant increase in nitrobacteria in the gastric mucosa of GC patients. The nitroproducts produced by these bacteria may promote tumor development by enhancing host cell genomic toxicity and other mechanisms (20). The analysis of metabolic functions in gastric flora of GC patients revealed an increase in lactic acid producing bacteria, enrichment of carbohydrate metabolism, and short-chain fatty acid metabolism (21). Researchers in the Narino region of Colombia, where *H. pylori* infection rates are high, studied stomach flora in coastal and mountainous populations with varying rates of GC (24). They identified specific flora in areas at higher risk of cancer. A recent study by Ling et al. focused on the imbalance of gastric flora and the development of an immunosuppressive microenvironment in GC tissues. The analysis identified a positive correlation between *Stenotrophomonas, Selenomonas*, BDCA2 positive dendritic cells, and Foxp3 positive Treg cells, suggesting a potential role of these bacteria in the formation of an immunosuppressive microenvironment in GC (53).

Epstein-Barr virus (EBV) plays a significant role in gastric carcinogenesis. In 1990, Burke et al. first identified EBV nucleic acid in undifferentiated lymphoid carcinoma (LELC) of the stomach using PCR (54). Subsequently, Shibata et al. confirmed EBV infection in gastric adenocarcinoma tissues through *in situ* hybridization (55). High levels of antibodies against EBV capsid antigens and early antigens were also found in the sera of GC patients with EBV infection. A 2014 molecular characterization analysis of EBVaGC, based on the TCGA GC cohort, revealed that 80% of this type of GC exhibited high frequency mutations of PIK3CA, high DNA methylation levels, hypermethylation of the CDKN2A promoter, and overexpression of JAK2, PD-L1, PD-L2, and ERBB2. Additionally, alterations in PTEN, SMAD4, and ARID1A were observed (56). EBV non-coding RNA (EBERS) was found to contribute to the downregulation of the miR-200 family, leading to reduced E-cadherin expression, a crucial step in EBV-associated carcinogenesis (57). Despite these findings, the specific pathogenesis of EBV in GC remains unclear.

Kumata et al. discovered human herpesvirus 7 (HHV-7) in the human stomach using macrotranscriptomics. Subsequent analysis revealed that the transcriptome of HHV-7 was consistent with the presence of resting memory CD4 T cells (57). This led to the hypothesis that HHV-7 may play a role in the development of GC. A study involving 45 cases of GC found a notable increase in *Candida albicans* and a decrease in *Saitozyma* and *Thermomomyces* in GC patients compared to controls (58). Zhu et al. shed light on the connection between dysregulation of mold populations, immune regulation, and tumorigenesis in the mouse gut. They reported that *Candida albicans* triggers the secretion of inflammatory IL-7 and leads to a metabolic shift to glycolysis through subcutaneous macrophages, ultimately inducing IL-22 secretion from innate lymphocytes and promoting tumor growth (59). Overall, these findings suggest that other bacteria may influence gastric carcinogenesis, but unlike *H. pylori*, their specific role remains speculative and requires further investigation.

## 3 Intestinal flora and TME

**Figure 2:**
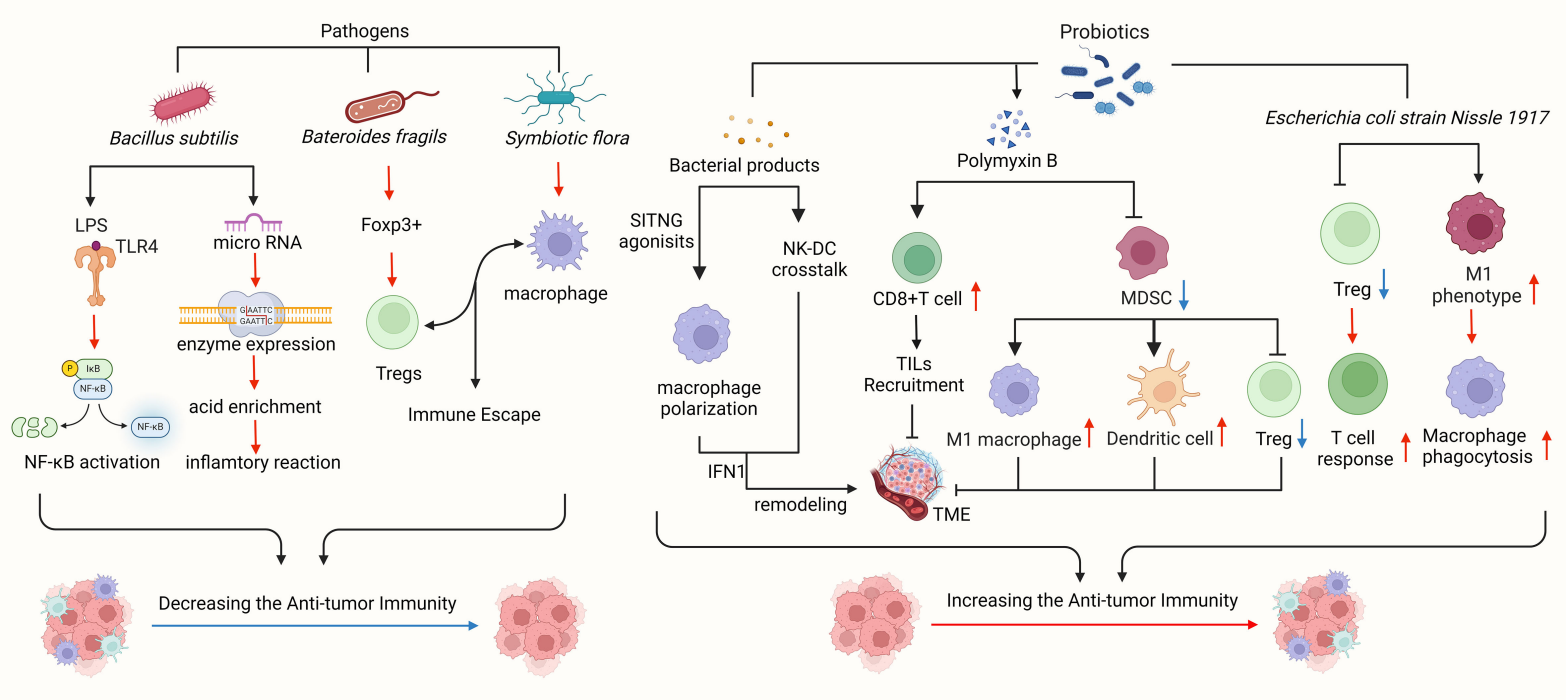
Intestinal flora plays a significant role in the tumor microenvironment. Pathogens linked to gastric cancer, such as *Bacillus subtilis, Bacteroides fragilis*, and *symbiotic flora*, can impede immune cell function, facilitate tumor immune evasion, and activate tumor-related signaling pathways to advance tumor growth. *Clostridium nucleatum* has been shown to enhance chemotherapy resistance in colorectal cancer by modulating TLRs, microRNA, and autophagy networks. *Bacteroides fragilis*, known for inducing Foxp3, a key mediator of gastrointestinal immunity and peripheral tolerance, can prompt IL-10-mediated mucosal tolerance, leading to the generation of Tregs, while commensal microorganisms aid in pDCs escape. Probiotics and bacterial metabolites can stimulate a robust immune response, bolster anti-tumor immunity, and impede tumor advancement. STING agonists from the microbiota trigger IFN-I production by mononuclear phagocytes in tumors, fostering an anti-tumor microenvironment. By modulating natural killer cell-DC communication, adjusting IFN-I levels, reshaping the tumor microenvironment, and enhancing response to immune checkpoint blockade. Polymyxin B hinders MDSC, diminishes DC and macrophage functions, and boosts Treg cell activity. *Bifidobacterium* directly prompts DC maturation and encourages T cells to mount immune responses. *Bacillus fragilis* induces macrophage polarization towards the M1 phenotype and enhances macrophage phagocytosis.

Tumorigenesis is a multifaceted process influenced by the interplay between tumor cells and TME, encompassing initiation, progression, and metastasis (60). The key components of TME consist of vascular cells, mesenchymal stem cells, tumor-associated fibroblasts, immune cells, and extracellular matrix (61, 62). Immune cells within TME include T cells, B cells, natural killer (NK) cells, dendritic cells (DC), tumor-associated macrophages, and other innate immune cells. Tumor cells can modulate the local microenvironment through the release of extracellular signals, promotion of angiogenesis, and induction of peripheral immune tolerance, while the components within the microenvironment can impact the growth and development of tumor cells (63). In recent years, advancements in second-generation sequencing technology and cell culture techniques have shed light on the relationship between the intestinal microbiota and malignant tumors. Some studies have demonstrated a close association between the intestinal microbiota, TME, and malignant tumors (**Figure 2**).

Intestinal flora can enhance the therapeutic effects of CpG oligonucleotides, platinum compounds, and cyclophosphamide by modulating the function of immune cells in TME, influencing immune cell composition, and boosting the activity of tumor-infiltrating effector T cells (64–66). *Bifidobacterium* has been shown to protect melanoma by supporting anti-PD-L1 therapy (67). Preclinical models suggest that the composition of gut microbiota may improve the efficacy of PD-L1 therapy by impacting TME (68). Depletion of gut bacteria can lead to immunogenic changes in the pancreatic ductal adenocarcinoma (PDAC) microenvironment, including increased differentiation of myeloid-derived suppressor cells (MDSCs) and M1-like tumor-associated macrophages (TAMs), as well as the activation of CD4+ Th1 and CD8+ T cells, which can enhance the effectiveness of checkpoint immunotherapy by increasing PD-1 expression (67). IL-25 has been found to activate M2 TAMs, induce the expression of chemokine CXCL10, and promote epithelial-mesenchymal transition (EMT) in hepatocellular carcinoma (HCC), thereby facilitating tumor progression and metastasis (69). Researchers have observed that depletion of intestinal flora leads to an increase in Th1 (INFγ)+CD4+CD3+ and Tc1 (INFγ)+CD8+CD3+ cells, while the number of IL-17A and IL-10-secreting cells decreases (70). Alterations in gut flora can stimulate the production of cytokines such as IL-17, IL-22, IL-6, GM-CSF, and TGF-β, which can shape TME, enhance the recruitment of MDSCs and tumor-promoting cells to TME, maintain the immune microenvironment, and support tumor immune surveillance through INF-γ and IL-17 (71).

Different intestinal flora may play varying roles in TME (67). Escherichia coli strain Nissle 1917 (EcN) has been identified as a positive regulator of TNF-α in tumor infiltrating lymphocytes (TILs), leading to the inhibition of tumor growth (64). Some studies have shown that a combination therapy involving a TGF-β blocker and EcN could effectively induce tumor suppression and necrosis in subcutaneous tumor-bearing mice, with superior therapeutic outcomes compared to using a TGF-β blocker or EcN alone. This enhanced therapeutic effect was associated with an increase in tumor-specific effector T cell response, tumor antigen-specific IFN-γ production, and an accumulation of CD8+ T cells (64). Researchers have also observed a correlation between colorectal cancer (CRC)-associated gut microbiota and the expression of the CXCR2 signaling gene, suggesting a potential mechanistic link between CXCR2 signaling gene expression and alterations in specific gut microbiota (72).

TME becomes increasingly hypoxic with larger tumor sizes, leading to anaerobic dominance in the gastrointestinal (GI) tract (73). Changes in intestinal flora can trigger the production of various cytokines and growth factors, ultimately influencing the recruitment of myeloid-derived suppressor cells (MDSC) and regulatory T cells (Treg) to TME (71). *Bifidobacterium*, a symbiotic anaerobic bacterium and a key component of gut flora enhances programmed death-ligand 1 (PD-L1)-based immunotherapy by improving dendritic cell (DC) function. While *Bifidobacterium* alone may not directly inhibit tumor growth systemically, it can boost the antitumor effects in mice unresponsive to CD47 blockade (73). A decrease in the diversity of tumor-associated microflora is associated with reduced expression of tissue-protective innate inflammatory signaling receptors like Toll-like receptors (TLR2, TLR5), and nucleotide-binding oligomerization domain (NOD) proteins (NOD1, NOD2). Dysregulation of these inflammatory signals promotes the growth of inflammatory and DNA-damaging bacterial species, contributing to the formation of tumor inflammatory microenvironments and influencing tumor progression (74). Researchers have also observed a positive correlation between the clinical efficacy of programmed cell death protein 1 (PD-1)/programmed death-ligand 1 (PD-L1) antibodies and the diversity of intestinal flora, as well as the presence of effector T cells in TME. Therefore, manipulating the composition of TME and intestinal flora could represent a promising strategy to enhance the clinical benefits of PD-1/PD-L1 antibody therapy (66).

## 4 Mechanism of intestinal flora regulating TME

Recent studies have provided a deeper understanding of the complex interrelationships between the gut microbiota and TME, but the mechanisms of how both regulate tumor growth are not fully understood. We believe that intestinal flora can promote or inhibit tumor growth by producing bacterial products and interacting with TME through pattern-binding receptors.

### 4.1 Bacterial metabolites

Gene products and metabolites from the gut flora play an important role in mediating immune responses and modulating inflammation, contributing to intestinal homeostasis (75–77). Short-chain fatty acids (SCFAs), such as butyric acid, are known to regulate both innate and adaptive immune cell functions, exerting anti-inflammatory effects by inhibiting immune cell recruitment and pro-inflammatory activities (78). In GC, gut microbiota-derived butyrate enhanced CD8+ T cell cytotoxicity via GPR109A/HOPX, thereby inhibiting tumor progression (79). Lipopolysaccharide (LPS), a product of Gram-negative bacteria in the gut, is elevated in the blood and tissues of patients with gastrointestinal neoplasms (80). A study found that LPS mediates the crosstalk between primary GC cells and the intrahepatic microenvironment by promoting TGF-β1 secretion in intrahepatic macrophages via interacting with LPS binding protein (LBP), which induces intrahepatic fibrotic pre-metastatic niche formation to promote GC liver metastasis (81). These findings suggest that clearing LPS from tumors could alleviate TME immunosuppression and improve the efficacy of immunotherapy.

Living bacteria actively migrate and continuously produce secondary metabolites, activating the stimulator of interferon genes (STING) pathway in dendritic cells. The intestinal flora alters TME through STING signal transduction at the tumor site, leading to an anti-CD47 immunotherapy effect (73). A study shows that knocking-down STING can promote TAMs polarizing into pro-inflammatory subtype and induce apoptosis of GC cells, mechanistically through IL6R-JAK-IL24 pathway (82). Pathogenic bacterial flora triggers the expression of the inflammatory enzyme COX-2 in gastrointestinal tract. Both COX-2 and COX-2-derived PGE2 have been shown to increase CXCR2 ligand levels in gastrointestinal tract tumors (72). COX-2 expression is elevated in GC and its precursor lesions. COX-2 expression is associated with reduced survival of GC patients, and it has also been shown to be an independent factor of poor prognosis. The regulation of COX-2 expression in GC cell lines involves a variety of molecular mechanisms, including the signal transduction pathway activated by *Helicobacter pylori*. In animal models of GC, the role of COX-2 seems to be mainly to promote tumor promotion and growth (83).

### 4.2 Pattern recognition receptors

Intestinal flora primarily functions through microbe-associated molecular patterns (MAMPs) and pathogen-associated molecular patterns (PAMPs). These patterns recognize and initiate immune signaling events on host cells via PRRs, including toll-like receptors (TLRs) located in cell membranes and nucleotide-binding oligomerization domain-like receptors (NLRs) located in the cytoplasm (84).

TLRs are potent proinflammatory stimulators that identify MAMPs such as lipopolysaccharides (LPS), peptidoglycans, flagella, and microbial DNA/RNA (85). Activation of TLRs leads to the activation of the nuclear factor-kappa B (NF-κB) signaling pathway, which is crucial for the expression of genes that regulate innate immunity and inflammation (86). Bacterial products recognized by TLRs, such as LPS, short-chain fatty acids (SCFA), and peptide chains, can activate the IL-23/IL-17 axis and promote the development of tumor (CRC) (70, 71). As one of PRRs, TLRs play a key role in the gastrointestinal innate immune response, and their signaling has been implicated in the pathogenesis of GC. The core adapter myeloid differentiation factor-88 (MyD88) is shared by most TLRs and functions primarily in *H. pylori*-triggered innate immune signaling. TLR/MyD88 signaling can manipulate the expression of infiltrating immune cells and various cytokines in the TME, thereby affecting the invasion and migration of GC (87).

### 4.3 Other mechanisms

Intestinal flora plays a significant role in influencing immunotherapy by impacting metabolic reprogramming, immune reprogramming, and immune cell reprogramming within TME, ultimately leading to changes in TME (88, 89). Previous research has demonstrated that tumor metabolic reprogramming is linked to tumor immune evasion. For example, the production of lactic acid through glycolysis can promote tumor cell metastasis, while oxidative compounds produced by tumor cells can lead to T cells metabolizing tryptophan into kynurenine, thereby inhibiting T cell activation in an immunosuppressive microenvironment (90). Ling et al. conducted a study comparing the gastric mucosal microbiota in samples from 59 GC patients, 60 normal individuals, and 61 peritumoral tissues (53). Their findings, analyzed through immunohistochemistry and Pearson correlation analysis, indicated that SBDCA2 pDCs and Foxp3+ Tregs play a role in modulating gastric mucosal flora and contributing to an immunosuppressive microenvironment in GC. Furthermore, microbiota-derived stimulator agonist IFN has been shown to reshape TME by influencing macrophage polarization and modulating NK cell-DC interaction (91).

MicroRNAs (miRNAs) play an important role as transcriptional regulators in a range of physiological processes, including immunity and metabolism. At present, it is believed that miRNAs play an important role in the pathogenesis of GC. In addition, miRNAs can be used as biomarkers of GC as well as therapeutic targets (92).

Thakkar et al. utilized a unique custom computational pipeline to analyze and describe bacteria in low-microbial-content endoscopic samples, comparing the microbiota of 15 GC samples with their adjacent non-cancerous mucosal tissues (93). The study revealed a specific enrichment of organisms such as *Veilonella parvula* (12/15) and *Prevotella melaninogenica* (10/15). Tumor-associated immune cells like Th1 and Th2 helper cells and macrophages showed a stronger presence in the tumor samples. Through ELISA analysis, elevated expression of proinflammatory cytokines (such as TNFα, IL-8, GRO, MCP-1, and IL-1a) was observed in tumor samples compared to normal mucosa. These findings suggest that the intestinal flora plays a role in mucosal immune alterations and the development of a proinflammatory TME.

In conclusion, further investigation is necessary to explore whether specific gut flora can induce changes in host homeostasis, such as inflammatory responses, pathway stimulation, or immune responses, and potentially influence tumor development by interacting with TME for therapeutic and diagnostic purposes.

## 5 Tumor and flora exosomes

### 5.1 BEVs and tumors

#### 5.1.1 EBV definition

**Figure 3:**
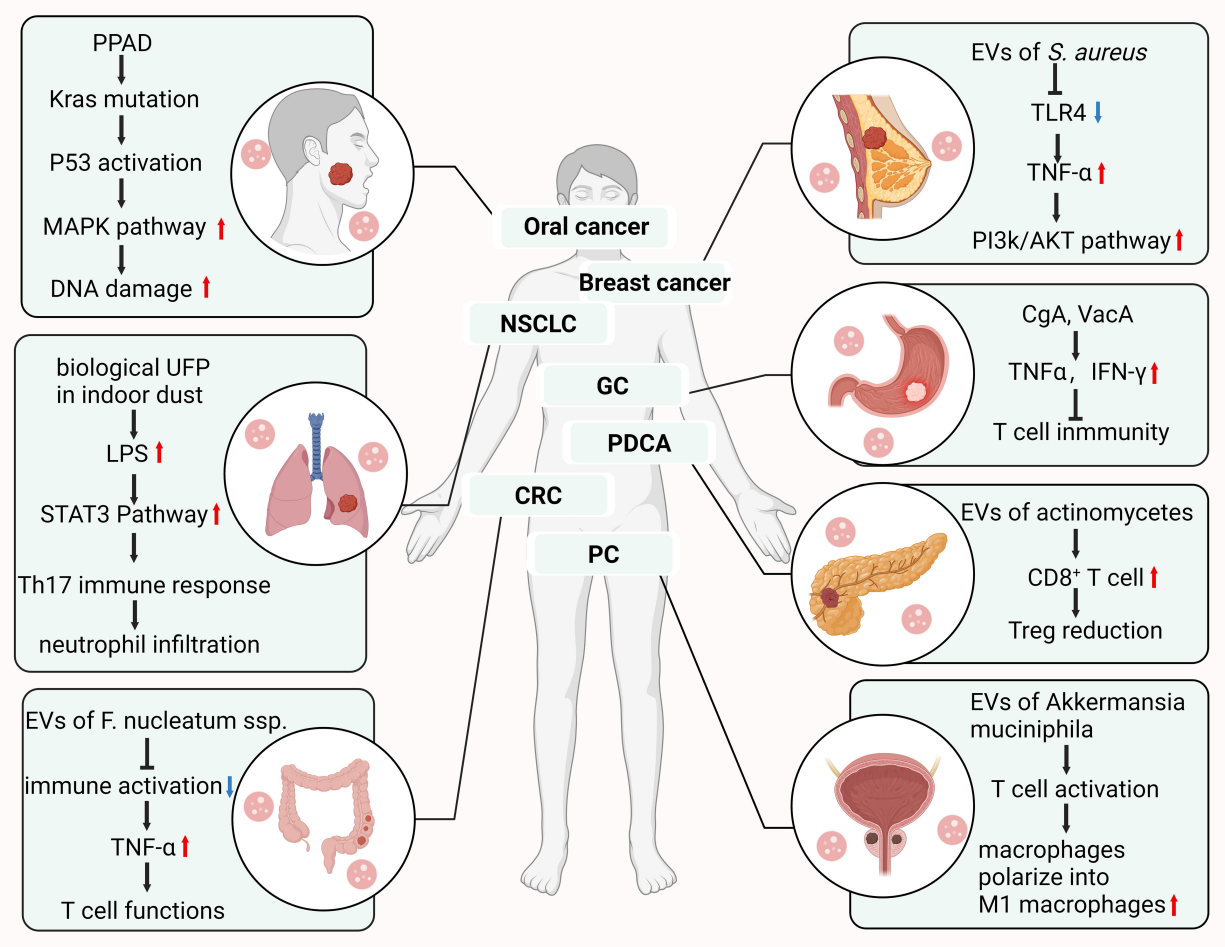
The mechanism of action of bacterial outer vesicles in different solid cancers.

In the context of understanding the role of intestinal microbial communities in human health and disease, microbial-derived extracellular vesicles (EVs) have emerged as a significant area of research. BEVs are a specific type of EVs released by bacteria. These spherical structures have a lipid bilayer and typically range from 20 to 400 nm in diameter. BEVs, as small molecule active substances derived from bacteria, offer advantages such as structural stability and prolonged circulation time (94). They are recognized as important mediators of interactions among bacteria and between bacteria and the host, influencing various physiological and pathological processes in both bacterial and host organisms (95). These processes include the transport of virulence factors, biofilm formation, antibiotic resistance, and impact on host physiological and pathological functions (96). Consequently, there is a growing body of research investigating the mechanisms by which BEVs contribute to tumor initiation and progression (**Figure 3**), as well as exploring their potential applications in cancer diagnosis and therapy.

#### 5.1.2 BEVs regulate cancer initiation and progression

The imbalance of the intestinal microenvironment, characterized by intestinal flora imbalance and a large accumulation of immune cells, is a significant factor in cancer development. Studies have shown a high presence of *H. pylori*-derived BEVs in the gastric juice of patients with GC, as well as an abundance of vesicles in gastric epithelial cells labeled with Dil stain. These findings suggest that BEVs may infiltrate gastric mucosa and epithelial cells, potentially playing an important role in gastrointestinal cancer (8). Another study identified alterations in microbiota composition and the abundance of specific bacterial species in patients with gastrointestinal cancer. Elevated levels of *Enterococcus, Escherichia coli, Shigella, Klebsiella, Streptococcus, Peptostreptococcus, Firmicutes, Clostridium*, and *Bacteroides* have been associated with abnormal cell proliferation and the promotion of cancer growth through the release of toxins (such as *Bacillus fragilis* extracellular vesicles) or by fostering inflammation and cancer cell proliferation (97).

BEVs derived from *H. pylori*-infected host cells influence inflammatory signaling pathways, thereby impacting cell proliferation, apoptosis, cytokine release, modification of immune cells, and endothelial dysfunction. Additionally, they disrupt cellular junctional structures, induce cytoskeletal reorganization, and play a crucial role in shaping subsequent immunopathological responses. These factors interfere with the pathogenesis of GC and affect its progression (98). Additionally, *H. pylori* liberates vesicles, called outer membrane vesicles (*H. pylori*-OMVs), which contribute to atrophic and cell transformation in the gastric epithelium (99).

Some bacterial EVs can either promote cancer progression or induce apoptosis of tumor cells, exerting antitumor effects. Beyond directly triggering apoptosis in colon cancer cells, BEVs can also serve as potent immunostimulators to stimulate antitumor immune responses *in vivo* for cancer treatment. OMVs released by bacteria have been shown to induce anti-BFGF autoantibodies in tumor-bearing mice, which inhibit tumor angiogenesis, enhance tumor cell apoptosis, reverse tumor immunosuppressive microenvironments, boost CTL responses, and ultimately impede tumor growth (100). *H. pylori* EVs are abundant in the gastric juices of GC patients. A study found that *H. pylori* EVs can induce inflammation and possibly cancer in the stomach, mainly via the production of inflammatory mediators from gastric epithelial cells after selective uptake by the cells (101). In addition, research by Li et al. shows that HSP60 derived from BEVs plays an important role in the progression of *Helicobacter pylori* related GC (102).

## 6 Bacterial extracellular vesicles in TME

### 6.1 BEVs reshape TME

BEVs play a critical role in regulating TME by influencing differentiation signals of immune cells and the release of immune cells and tumor substances. Recent research indicates that BEVs are involved in reshaping TME, potentially impacting cancer progression, metastasis, drug resistance, and immunosuppression. Ma Guanghui’s team proposed utilizing OMV nanoparticles as a platform for tumor immunotherapy. They achieved safe and effective regulation of the tumor immune microenvironment by coating OMVs with a calcium phosphate (CaP) shell using a biomimetic mineralization method. Upon reaching the tumor site with nanometer-sized particles, the acidic environment caused the calcium phosphate shell to dissolve, exposing the OMVs. This exposure effectively enhanced the tumor immune suppression microenvironment by facilitating the infiltration of cytotoxic T cells and the polarization of M2 macrophages to M1 (103). Feng et al. introduced a controllable bidirectional adapter based on OMV (OMV-CD47nb) that activates TAM phagocytosis of tumor cells through various pathways, such as inducing M1 polarization and blocking ‘don’t eat me’ signals. Furthermore, TAM activation stimulates T cell-mediated anti-tumor immunity by enhancing antigen presentation. The formulation also includes radiation-triggered controlled release of OMV-CD47nb, leading to TME remodeling upon radiation of tumors in mice injected with the nanoformulation (104). Guo et al. developed a co-delivery system for chemical drugs and Redd1-siRNA using bacterial outer vesicles as carriers. They also utilized mannose modification to improve the targeting of extracellular vesicles to M2 macrophages. Their study in breast cancer models revealed macrophage activation, tumor immunity activation, and modifications in TME. This demonstrates the targeted regulation of various cell types within TME through the bacterial outer membrane vesicle delivery system (105). Additionally, non-repressible Amuc_2172 led to H3K14ac up-regulation at the Hspa1a locus, stimulating the transcription and secretion of heat shock protein 70 (HSP70) in colorectal cancer cells. This process promoted a CD8 cytotoxic T lymphocyte (CTL)-related immune response in colorectal cancer (CRC) and reprogrammed TME (106). Bacterial outer membrane vesicles possess a unique structure and immunostimulatory effects that can induce tumor regression by activating the host immune system and reversing the immunosuppressive TME. In GC, EV-derived HSP60 reshapes TME and affects tumor progression (102).

### 6.2 BEVs regulate TME immunogenicity mechanism, regulate immune cells, and then anti-tumor

TME is a complex ecosystem where immune cells like NK cells, macrophages, T cells, and B cells can interact with tumor cells, leading to changes in their functions and characteristics that influence tumor evolution. The interaction between extracellular vesicles from specific gut microbiota and TME plays a role in the anti-tumor effect. Research indicates that non-toxic outer membrane component-free nanovesicles (PDNV) can be produced by genetically modifying bacterial protoplasts. These nanovesicles can target tumor tissue specifically and deliver chemotherapeutic drugs. The method involves the continuous compression of nano-size membrane filters to prepare nanovesicles with tumor-targeting moieties on the surface. These nanostructures enable passive targeting, while the presence of tumor-targeting moieties enhances tumor-specific uptake through receptor-mediated targeting (107). Bacterial extracellular vesicles (BEVs) can enhance anti-tumor immunity by interacting with follicular helper T cells (Tfh). For instance, treatment with *E. coli* outer membrane vesicles (OMV) can boost the infiltration and activation of CD8+ T cells, particularly cancer antigen-specific CD8+ T cells expressing high levels of TCF-1 and PD-1. Moreover, combining *E. coli* OMV with anti-PD-1 antibody immunotherapy can effectively inhibit tumor growth and activate cancer antigen-specific stem cell-like CD8+ T cells. These findings suggest that *E. coli* OMV could serve as a promising cancer immunotherapy agent with potent anti-tumor properties (108). Engineered OMV-PD1 binds to PD-L1, facilitating its internalization and degradation, thereby shielding T cells from the immunosuppressive PD1/PD-L1 axis. Through a dual mechanism of immune activation and checkpoint inhibition, engineered OMV drives the accumulation of effector T cells within tumors (109). Researchers developed a novel nanovesicle (HNVs) derived from three intestinal bacterial strains associated with positive responses to immune checkpoint therapy. Comprised mainly of bacterial cell membrane proteins and devoid of pyrogenic substances, HNVs have demonstrated superior tumor and lymphoid organ targeting capabilities. Additionally, they induce innate immune activation, promote dendritic cell maturation and antigen presentation, and enhance TME. In mouse models of pancreatic cancer, combining HNVs with α PD-1 therapy effectively suppresses tumor growth, induces innate immune activation, inhibits tumor oxidative phosphorylation, remodels the tumor immune microenvironment, and enhances therapeutic outcomes (110). Wang et al. engineered a hybrid membrane consisting of bacterial outer membrane vesicles (OMV) and B16-F10 cancer cell (CC) membrane, encapsulated within hollow polydopamine (*H. pylori* DA)NP. By combining OMV immunotherapy with *H. pylori* DA-mediated phototherapy (PTT), they enhanced the antitumor efficacy against melanoma. Injecting OMV-CC of (*H. pylori* DA)NP into the tail vein of tumor-bearing animals resulted in uniform melanoma targeting, dendritic cell maturation in lymph nodes, and immune response activation. The findings indicate that the combination of anti-tumor immune response and PTT significantly improves therapeutic efficacy and leads to complete melanoma elimination (111). Bacteria-plant hybrid vesicles (BPN) and OMV can target tumor tissues, activate immune cells, and release tumor-associated antigens, leading to the stimulation of tumor-specific CD8+ T cell responses (112). Additionally, the administration of OMV has been found to elicit epitope-specific T-cell responses and inhibit tumor growth. Studies have demonstrated that the use of BPN and OMV in tumor tissues can effectively induce tumor-specific T-cell responses, thus laying a crucial foundation for the development of novel anti-cancer immunotherapies (113). Overall, these vesicles, produced by modified microbial communities, play a role in modulating tumorigenesis by interacting with immune cells through mechanisms that enhance the immunogenicity of TME.

### 6.3 BEVs directly enter TME and release various metabolites affecting TME

Various studies have demonstrated that bacteria release bioactive metabolites through extracellular vesicles, which can selectively accumulate around tumor cells and alter TME (114, 115). For instance, *E. coli* has been shown to generate loop regulatory proteins and gene toxins, leading to chromosome instability and DNA damage that can facilitate the onset of colorectal cancer (116). Moreover, Che et al. discovered that vesicles released by GC cells infected with *H. pylori* contained virulence factors such as cytotoxin-related gene A and vacuolar cytotoxin A, which could stimulate macrophages to produce multiple cytokines (TNF-α, IL-6, and IL-1β), thereby supporting the progression of GC (101). Consequently, it is speculated that the metabolites produced by these extracellular vesicles interact with TME, potentially disrupting the normal regulation of genetic material in host intestinal cells and contributing to tumor development.

### 6.4 BEVs regulate TME through inflammatory mediators

There is clear evidence that inflammation is a high-risk factor for many malignant tumors, and participates in many processes such as malignant transformation, tumor formation, development, invasion, and metastasis (117). The presence of inflammatory carcinogenic metabolites in cancer cells is usually attributed to interactions between TME and resident microbiota (8). The researchers found that *H. pylori* EV is abundant in the gastric juice of GC patients, mainly through selective uptake of gastric epithelial cells to produce inflammatory mediators, which can induce stomach inflammation and possible cancer (101).In addition, *L. paracasei*-derived extracellular vesicles attenuate LPS-induced intestinal inflammation through endoplasmic reticulum stress activation and may play a significant role in maintaining colorectal homeostasis in inflammation-mediated pathogenesis (118).Further studies have shown that some intestinal bacteria can release toxin-infected EV, which promotes the development of colorectal cancer by exacerbating inflammatory conditions. For example, the fragilis toxin secreted by *Bacteroides fragilis* into EV can promote colon tumor growth, mediate E-cadherin cleavage and IL-8 production (119). Choi et al. found that BEVs induces interferon gamma, IL-17 and EV-specific immunoglobulin *in vivo* in mice, which shape inflammatory TME and promote gastric cancer progression (101). Therefore, we hypothesize that BEVs play a key role in the transition from inflammation to cancer.

## 7 Repurposing BEVs in medical applications

### 7.1 BEVs as vaccines

BEVs are not replicative but highly immunogenic and can be utilized as vaccines, adjuvants, and vectors for treating diseases, particularly in presenting tumor antigens or small molecule drug targeted therapies. Initially used as adjuvants to enhance the immune response of meningitis type B (MenB) vaccines, OMVs may also contain pathogen-specific antigens (120). For example, *Escherichia coli* OMV with an L2 repeat sequence can trigger the production of anti-HPV functional antibodies, offering an effective and cost-efficient method for developing a universal anti-HPV vaccine (121). Additionally, OMVs have demonstrated anti-tumor activity by specifically accumulating in tumor tissues after intravenous injection and inducing an anti-tumor immune response, ultimately leading to tumor eradication (122).

Ping et al. developed a strategy to co-deliver outer membrane vesicles (OMV) and chemotherapeutic drugs to tumors. They first modified detoxifying OMV with polyethylene glycol (PEG) and the tumor targeting ligand Arg-Gly-Asp (RGD) peptide to enhance blood circulation and tumor targeting. Subsequently, they coated OMV on tegafur-loaded nanoparticles, a chemotherapeutic drug that sensitizes cancer cells to T cells and removes immunosuppressive myeloid suppressor cells (MDSCs). The resulting OMV-coated nanoparticles demonstrated the ability to target tumors (122). Most vaccine formulations require adjuvants to boost the immune response. Tamoxifen has been shown to enhance the therapeutic effect against breast cancer when combined with extracellular vesicles (EVs) of *Staphylococcus aureus*. This suggests that *Staphylococcus aureus* EVs could potentially be used as adjuvants in breast cancer treatment in the future (123). It is anticipated that a significant number of highly effective and low-toxic OMV vaccines will progress to clinical trials in the near future.

### 7.2 BEVs as delivery vehicles

Due to their small size and natural lipid bilayer, bacterial extracellular vesicles (BEVs) can encapsulate a variety of biomolecules, including lipids, nucleic acids, and proteins, making them promising drug delivery systems. Kim et al. (2017) demonstrated that intravenous administration of EVs loaded with engineered bacteria can trigger potent and durable IFN-γ and T cell-mediated anti-tumor immune responses, leading to tumor eradication (122). A novel ‘plug-and-play’ approach involving the modification of programmed death protein-1 (PD-1) on the outer membrane of BEVs has been developed to enhance PD-L1 binding to tumor cells while safeguarding T cells from the PD-1/PD-L1 immunosuppressive pathway (124). This personalized tumor therapy strategy based on BEVs allows for the customization of tumor antigens for individual patients, enabling the design of BEV vaccines expressing specific tumor antigens (125). Notably, the epitope TRP2180–188 of melanoma tyrosinase-related protein 2 (TRP2) has been successfully displayed on BEV surfaces using ‘plug-and-play’ techniques. In preclinical models, BEV immunization induced robust immune responses, reduced tumor growth, and inhibited metastasis (126). Furthermore, Wang et al. engineered Escherichia coli to secrete *H. pylori* V16 E7 protein into the periplasmic space of outer membrane vesicles (OMVs) using gene recombination technology (127). These modified OMVs facilitated the uptake and intracellular delivery of tumor antigens by dendritic cells, resulting in potent E7-specific cellular immune responses and significant suppression of tumor growth in mouse xenograft models.

Some Gram-negative bacterial outer membrane vesicles (GBEVs) have been shown to enhance the host immune system and can be utilized in combination with anticancer drugs to produce synergistic therapeutic effects. Researchers employed rigorous purification techniques to isolate bioengineered OMVs (AffiHER2 type) from impurities, which were then loaded with siRNA drugs through electroporation to enhance their inhibitory effects on tumor cells. Further studies revealed that targeting HER2 overexpressing tumor tissue allowed the siRNA carried by OMVs to be delivered specifically to tumor cells, resulting in an anti-tumor effect (128). Coating bacterial outer membrane vesicles on polymer micelles loaded with fluorouracil has been demonstrated to sensitize cancer cells to cytotoxic T lymphocytes (CTL) and induce direct cancer cell death, thereby inhibiting tumor growth and metastasis *in vivo (* 107). Additionally, Kim et al. developed protoplast-derived nanovesicles (PDNV) from bacteria expressing epidermal growth factor (EGF), which can target chemotherapy drugs to selectively kill cancer cells (129). Importantly, chemotherapy-loaded bacterial outer membrane vesicles increased doxorubicin infiltration and induced tumor cell apoptosis, suggesting a promising strategy for targeted drug delivery (130).

### 7.3 BEVs as diagnostic biomarkers

BEVs play an important role in bacterial communication and host immune regulation. The exchange of BEVs between pathogens during host damage not only serves as improved biomarkers for pathogenic processes, but also offers potential for monitoring local and systemic infections, making them valuable for diagnosing infectious diseases (131). BEVs are an emerging biomarker that can be detected through liquid biopsy, opening up new diagnostic possibilities for various diseases (132). Furthermore, clinical analysis of BEVs isolated from biological fluids of patients and healthy controls has identified two distinct groups. It is noteworthy that the diagnostic model developed using an external validation set has shown promising predictive capabilities for colorectal cancer, liver cancer, GC, pancreatic cancer, and biliary cancer (133).

Bacterial extracellular vesicles (BEVs) are vehicles that contain nucleic acids, proteins, and/or lipid molecules derived from parent bacteria, which are secreted during the life cycle of living bacteria. By detecting these molecules within BEVs, researchers can characterize changes in the intestinal microbiota and metabolites of patients more accurately, thereby identifying biomarkers. For instance, Zhang et al. analyzed the respiratory tract microbiome isolated from bronchoalveolar lavage fluid of lung cancer patients and detected *Veillonella dispar* as dominant in the high PD-L1 group, while the population of *Neisseria* was significantly higher in the low PD-L1 group (134). Similarly, Kim et al. found that BEVs isolated from stool samples of colon cancer patients showed a significant increase in the abundance of *Firmicutes (*e.g. *Eubacilli, Faecobacteria, Ruminococcaceae, and Lianobacteria)* compared to healthy controls (135). These results indicate that BEVs as biomarkers for GC may have potential in the future. However, research on disease-associated biomarkers in BEVs is still in its early stages, emphasizing the importance of effective isolation and detection of BEVs in biological samples, necessitating rapid and accurate review.

## 8 Conclusion and future perspectives

The intestinal microbiome plays an important role in influencing the progression and prognosis of GC by affecting TME. This review article provides an overview and comparison of the biological targets through which the intestinal microbiome regulates TME, laying the groundwork for potential applications in tumor prevention, diagnosis, treatment, and prognosis. While there is some understanding of how the intestinal microbiome impacts TME and the combined effects on tumor development, further in-depth research is needed. Several key challenges remain: Firstly, it is essential to consider the individual variations in intestinal flora between animal models and humans, and to explore the microbial composition of the intestinal flora in diverse individuals to develop personalized strategies for optimizing intestinal flora in precision medicine. Secondly, the identification of specific bacterial components and clarification of how metabolic pathways influence TME are pressing issues that require further investigation, along with understanding their mechanisms of action. Lastly, exploring whether the intestinal flora and TME can serve as promoters of tumor immunotherapy, and determining how to manipulate gut microbiota and TME to enhance the efficacy of immunotherapy in cancer patients, improve the effectiveness of immune checkpoint inhibitors, and devise new therapeutic approaches targeting TME for anti-tumor activity are critical for advancing gut microbiota-targeted therapies that could enhance the outcomes of current cancer treatments.

Recent research has discovered that intestinal flora communicates between cells through the release of extracellular vesicles, opening up new avenues for investigation. These vesicles may play an important role in interactions between intestinal microorganisms and hosts, as well as in immune regulation. However, the review does not clearly specify the specific patient populations that could benefit from combined therapy involving intestinal microorganisms and BEVs. While BEVs show promise in the prognosis, diagnosis, and treatment of various diseases, there are limitations to consider. These include the potential accumulation of EV or bacterial toxins in the host, the high cost and complexity of EV purification and isolation, low levels of protective antigen expression, and the potential interference with immune responses by immunosuppressive molecules. Moreover, the current evidence mainly comes from preclinical studies. There is a lack of direct clinical studies confirming the ability of intestinal microbes and BEVs to induce anti-tumor effects through the regulation of intestinal flora. Future research and applications should give sufficient attention to these limitations.

This article systematically reviews the biological targets of gut microbiota in regulating TME, laying the foundation for its potential applications in tumor diagnosis, treatment, and prognosis. However, the article lacks a detailed outlook on future applications, which is a major flaw.

## Author contributions

MY: Writing – original draft.
